# Video-assisted thoracoscopic right upper lobectomy in a patient with a right-sided aortic arch and Kommerell diverticulum

**DOI:** 10.1186/s12957-018-1477-4

**Published:** 2018-08-30

**Authors:** Chen Huang, Xunyu Xu, Qianshun Chen, Shengmei Lin

**Affiliations:** 1 Department of Thoracic Surgery, Fujian Provincial Hospital, Provincial Clinical College of Fujian Medical University, No. 134 East St., Fuzhou, 350001 Fujian China; 2 Department of Radiology, Fujian Provincial Hospital, Fuzhou, 350001 China

**Keywords:** Right-sided aortic arch, Video-assisted thoracic surgery, Lobectomy, Mediastinal lymph node dissection

## Abstract

**Background:**

It is a very rare condition for a patient to have right lung cancer and a right-sided aortic arch simultaneously. Right lobectomy under video-assisted thoracoscopic surgery (VATS) in such a patient is a challenging procedure that is seldom reported. We successfully performed a VATS right upper lobectomy in a 77-year-old female with a right-sided aortic arch and Kommerell diverticulum.

**Case presentation:**

A 77-year-old woman was referred to our division for a mixed ground-glass opacity lesion in the right upper lung. A right-sided aortic arch with Kommerell diverticulum was identified by preoperative 3D CT reconstruction. A VATS right upper lobectomy with radical mediastinal lymph node dissection was performed, and the final histological staging was Ia3 (pT1cN0M0). The patient was discharged without any complications.

**Conclusions:**

We conclude that the video-assisted thoracic surgery can be safely performed in such conditions. It is difficult to determine the extent of upper mediastinal lymph node dissection in such cases.

## Background

Right-sided aortic arch with Kommerell diverticulum is a rare congenital vascular structure variation, with a reported incidence of approximately 0.1% [1]. In such patients, right lobectomy under video-assisted thoracoscopic surgery (VATS) is a challenging procedure that is seldom reported. Here, we report such a case and share our experience.

## Case presentation

**Fig. 1:**
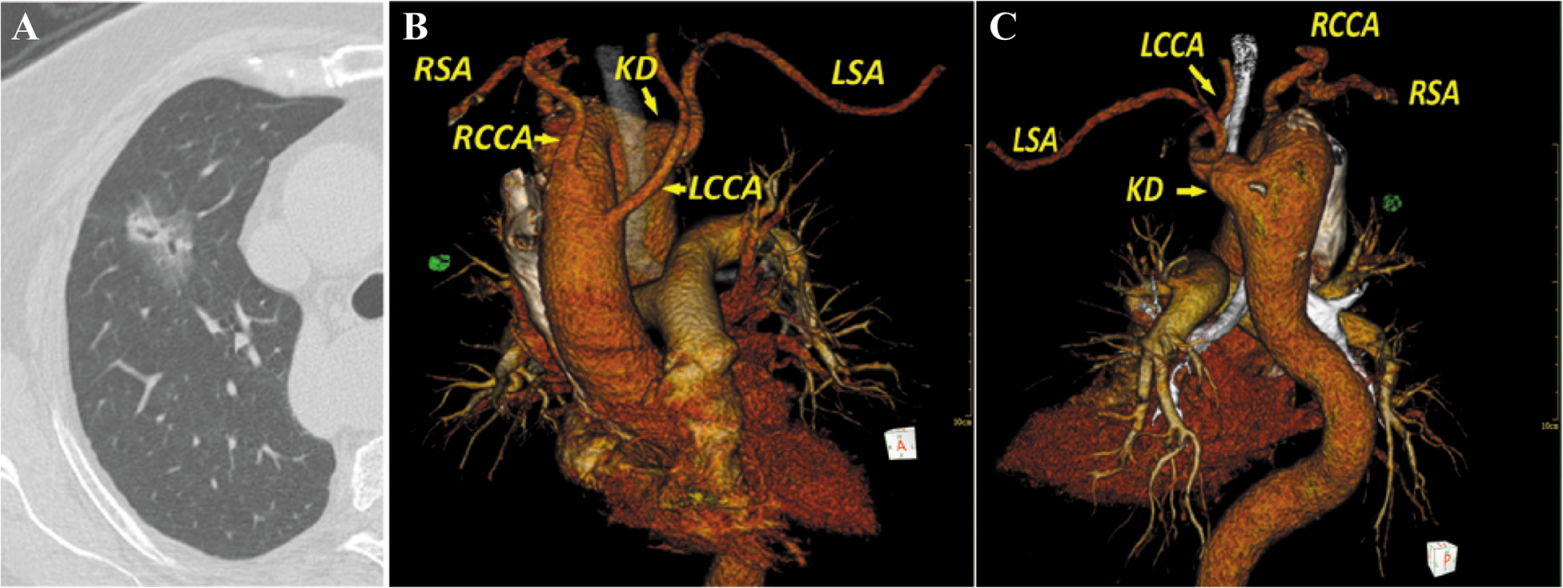
**a** Preoperative CT reveals a 34 mm × 20 mm partial solid nodule at the right S3. **b** The anterior view of the 3D CT image shows the right-sided aortic arch with the aberrant left subclavian artery. **c** The posterior view of the 3D CT image shows the Kommerell diverticulum of the left subclavian artery. RSA, right subclavian artery; RCCA, right common carotid artery; LSA, left subclavian artery; LCCA, left common carotid artery; KD, Kommerell diverticulum

A 77-year-old woman was referred to our division for a mixed ground-glass opacity lesion in the right upper lung. The patient was symptom-free and in good performance status. There were no remarkable findings on physical examination. Chest computed tomography revealed a 34 mm × 20 mm partial solid nodule with spicule formation and pleural indentation at the right S3 (Fig. 1a). The consolidation tumor ratio was 30%. Preoperative positron emission tomography-computed tomography (PET-CT) revealed that the standard uptake value (SUV) of the lesion was 1.9, and there was no suspicious metastasis. Kommerell diverticulum at the origin of the left subclavian artery and a right-sided aortic arch were detected through preoperative three-dimensional computed tomography (3D CT) reconstruction (Fig. 1b, c). Echocardiography revealed no cardiac abnormalities.

**Fig. 2:**
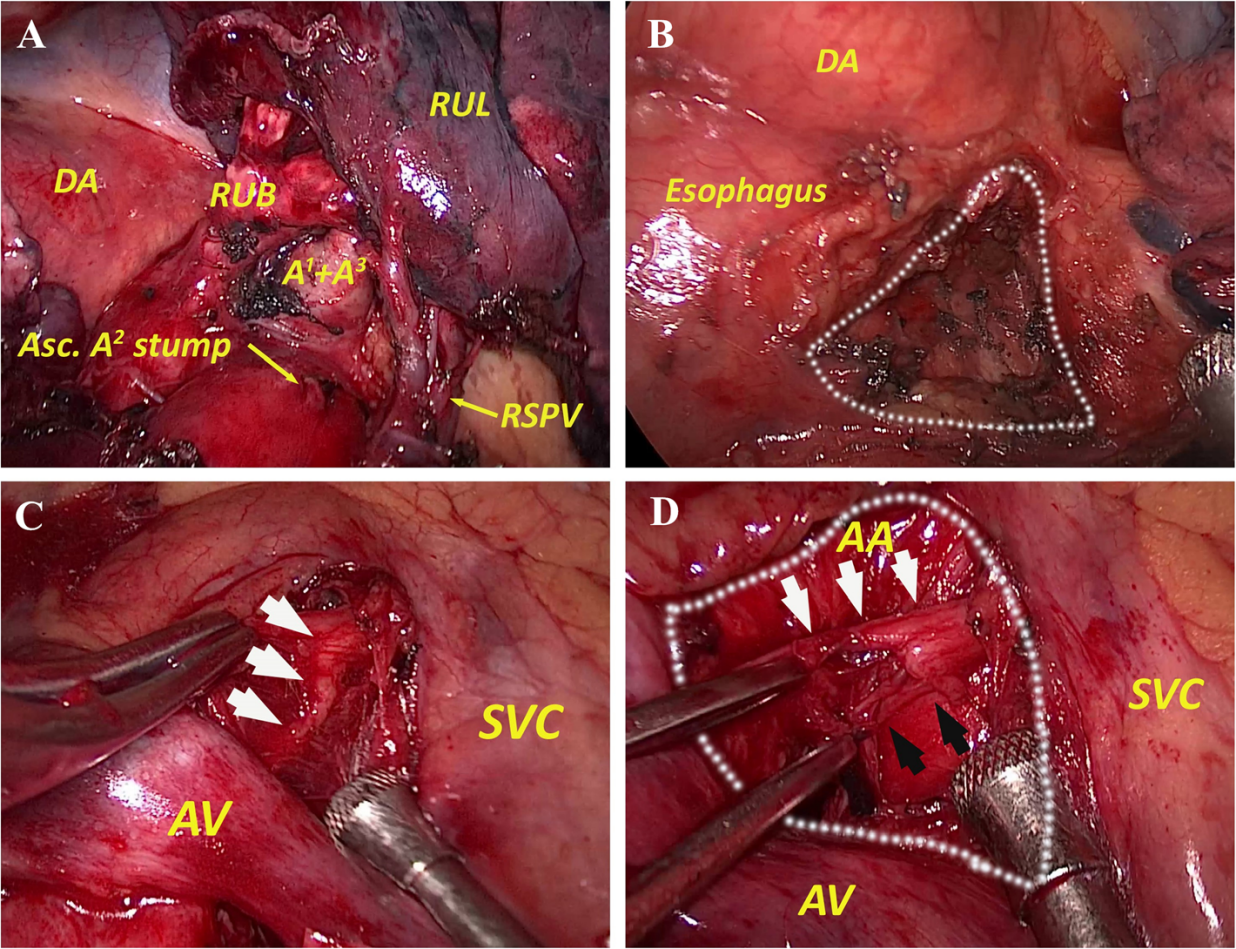
**a** The intraoperative view of the hilum structure shows there are no abnormalities of the pulmonary vein, artery, or bronchus. **b** The intraoperative view after the dissection of the subcarinal lymph node (#7). The dotted line shows the extent of the #7 lymph node. **c** The right vagus nerve (white arrow) is detected above the azygos vein. **d** The intraoperative view after dissection of the lower paratracheal lymph node (#4). The right RLN (black arrow) is observed to branch up from the vagus nerve (white arrow) and hook around the right-sided aortic arch. The dotted line shows the extent of the #4 lymph node. AA, aortic arch; Asc, ascending; AV, azygos vein; DA, descending aortic; RUB, right upper lobe bronchus; RUL, right upper lobe; RSPV, right superior pulmonary vein; SVC, superior vena cava

A right upper lobectomy was performed because the lesion was considered to be malignant. A three-port VATS approach with no rib spreading (no soft tissue retractor or direct visualization) was used. A 3-cm incision in the fourth intercostal space (ICS) at the anterior axillary line was made as the main manipulation port, and a 1-cm incision in the seventh ICS at the scapular line was made as an assisted manipulation port. A 1-cm thoracoscopic port was made in the seventh ICS at the middle axillary line. After dividing the interlobar fissure with a linear stapler, we detached and divided the ascending *A*^2^, right superior pulmonary vein (RSPV), *A*^1^ + *A*^3^, and right upper lobe bronchus sequentially. There were no anatomic variations of pulmonary vessels or bronchi (Fig. 2a). After histologically confirming the invasive carcinoma, radical mediastinal lymph node dissection was performed. After the routine dissection of the pulmonary ligament lymph node (#9) and the paraesophageal lymph node (#8), we dissected the subcarinal lymph node (#7). It was difficult to expose the left main bronchus, as the esophagus could not be suspended easily due to the obstruction of the right descending aorta (Fig. 2b). When dissecting the paratracheal lymph node, we identified the right vagus nerve above the azygos vein by blunt separation, and we then proceeded with the dissection in the cranial direction (Fig. 2c). The right recurrent laryngeal nerve (RLN) was observed to branch from the right vagus nerve and hook around the right-sided aortic arch (Fig. 2d). No upper paratracheal lymph node was dissected because of the obstruction of the right-sided aortic arch. The total operative time was 170 min with an estimated blood loss of 100 cc.

The postoperative histological diagnosis was moderately differentiated adenocarcinoma (80% lepidic and 20% acinar). There were five lower paratracheal lymph nodes, five subcarinal lymph nodes, two paraesophageal lymph nodes, two pulmonary ligament lymph nodes, one #10 lymph node, and two #11 lymph nodes retrieved. No lymph node metastasis was detected. The patient was discharged on postoperative day 7 without any complications such as hoarseness.

## Discussion

Right-sided aortic arch is a rare congenital malformation. According to the classification of Stewart et al. [2], our case was type II, which accounts for 40% of cases. This type has an aberrant left subclavian artery that originates from the descending aorta and is rarely associated with congenital heart disease. The stem of the left subclavian artery is usually dilated, and this is called a Kommerell diverticulum (Fig. 1c).

There have only been eight cases in the past 15 years, including our case, with a right-sided aortic arch in which the patient underwent lobectomy for right lung cancer (Table 1) [3–7]. Only one surgery was performed under VATS [7]. To the best of our knowledge, this is the first case report in which right upper lobectomy was performed under VATS in a patient with a right-sided aortic arch. In our experience, lobectomy of the right upper lobe under VATS in such a case is different from that of the other two right lobes. We can easily divide the RSPV from the main manipulation port with a stapler in normal cases. In patients with a right-sided aortic arch, the right aortic arch will obstruct this procedure. Therefore, we dissected the RSPV from the assisted manipulation port.

**Table 1:** Summary of reported cases of patients underwent right lobectomy for cancer with a right-sided aortic arch

Case	Year	Authors	Age	Gender	Pathalogical type	Tumor location	Surgical approach	Stage	Stewart classification	Path of right RLN
1	2003	Suzuki et al. [3]	67	Female	Squamous carcinoma	Right middle lobe	Right thoracotomy	N/A	N/A	N/A
2	2006	Hara et al. [4]	61	Female	Adenocarcinoma	Right upper lobe	Right thoracotomy	N/A	II	N/A
3	2008	Nakanishi [5]	N/A	N/A	Squamous carcinoma	Right upper lobe	Right thoracotomy	T3N0M0	II	Hooked around the right aortic arch
4			N/A	N/A	Squamous carcinoma	Intermediate bronchus	Right thoracotomy	T2N2M0	II	Hooked around the right aortic arch
5	2009	Suehisa et al. [6]	61	Male	N/A	Right lung	Right thoracotomy	N/A	II	Hooked around the right aortic arch
6			69	Male	N/A	Right lung	Right thoracotomy	N/A	II	Hooked around the right aortic arch
7	2014	Kodate et al. [7]	57	Male	Small-cell carcinoma	Right lower lobe	VATS	T1aN0M0	II	Hooked around the right aortic arch

*RLN* recurrent laryngeal nerve, *N/A* not available, *VATS* video-assisted thoracoscopic surgery

Dissection of the upper mediastinal lymph nodes in patients with a right-sided aortic arch differs from the routine dissection because the aortic arch is overhanging the trachea. It is difficult to determine the extent of upper mediastinal lymph node dissection in such cases. We interpreted the lymph nodes between the aortic arch and azygos vein as lower paratracheal lymph nodes (#4) (Fig. 2d). We believe the upper paratracheal lymph node (#2) was not available in this case because the right-sided aortic arch was overhanging the trachea. Moreover, visualizing the RLN was another major difficulty. We identified the right vagus nerve above the azygos vein first and then proceeded with the dissection in the cranial direction to detect the right RLN. Similar to previous cases [3–7], the right RLN of this patient branched up from the vagus nerve and hooked around the right-sided aortic arch. Blunt separation and sharp separation were used as alternatives to dissect the paratracheal lymph node. It is important to pay attention to the path of this nerve to avoid postoperative hoarseness.

## Conclusions

VATS right lobectomy with mediastinal lymph node dissection can be safely performed in patients with a right-sided aortic arch. A magnified view by thoracoscopy is helpful to identify the RLN path during mediastinal lymph node dissection. It is difficult to determine the extent of the upper mediastinal lymph node dissection in such cases because the anatomical indicators for mediastinal lymph node dissection are different from normal cases.

## Abbreviations

3D CT: Three-dimensional computed tomography
ICS: Intercostal space
PET-CT: Positron emission tomography-computed tomography
RLN: Recurrent laryngeal nerve
RSPV: Right superior pulmonary vein
SUV: Standard uptake value
VATS: Video-assisted thoracoscopic surgery
